# Plasma senescence associated secretory proteins: A new link to mild cognitive impairment

**DOI:** 10.1016/j.jnha.2025.100561

**Published:** 2025-04-24

**Authors:** Reema Banarjee, Nathan Basisty

**Affiliations:** Translational Gerontology Branch, National Institute on Aging, NIH, 251 Bayview Blvd, Baltimore, MD 21224, United States

Cellular senescence is widely acknowledged hallmark of aging that has been implicated in the progression of several age associated disorders. The secretome of senescent cells, termed as senescence associated secretory phenotype or SASP, consists of a number of inflammatory cytokines as well as growth factors and proteases [1] that can lead to paracrine disruption of normal tissue structure and function and propagate senescence in neighboring cells. Moreover, many SASP molecules have been identified as potential biomarkers of aging and associated traits [[2], [3], [4]]. In cases of age associated neurodegenerative diseases that can lead to dementia or cognitive impairment, increased senescence has been observed in multiple cell types in brain. For instance, senescent astrocytes have been shown to accumulate in the mid-brain of subjects with Parkinson’s disease and studies in mouse models have shown that their removal can preserve cognitive function [5,6]. Similarly, elevated senescent cells has been observed among excitatory neurons in the brain of subjects with Alzheimer’s disease (AD) [7] showing greater accumulation of neurofibrillary tangles [8], suggesting that the senescent cells might play a role in the progression of AD. Particularly, glial cell senescence has been attributed to aggravation of neuroinflammation in AD via the SASP [9]. Interestingly, increased accumulation of SASP has also been observed in plasma of AD patients [10], suggesting the potential of SASPs as clinical biomarkers for AD and other forms of dementia.

Mild cognitive impairment is defined as a condition defined by cognitive impairment with minimal impairment of daily activities. It is observed in about 10–20 % of individuals over 65 years of age and about 10% of individuals with MCI can progress to dementia every year [11]. Timely diagnosis of MCI can be helpful to identify individuals at high risk for progressing to AD. Currently, its diagnosis is based on neuropsychological testing or clinical assessments that determine the absence of dementia such as Alzheimer’s disease. There are few potential plasma biomarkers that have been reported [12,13], particularly senescence-associated protein biomarkers. Therefore, there is a need to identify new robust potential plasma biomarkers that can be applied clinically for diagnosis of MCI. Interestingly, senescent PBMCs have been reported in patients with MCI, indicating that senescence could be involved in its progression [14] and suggesting potential value in SASPs as diagnostic biomarkers of MCI.

In the study by Mielke et al., the authors have explored this possibility by analyzing the plasma levels of certain SASP markers to predict risk of mild cognitive impairment (MCI) among older adults [15]. The study is based on the data from the Lifestyle Interventions for Elders (LIFE), a large cohort study designed to assess the effects of physical activity and health education on mobility in sedentary older adults [16]. The authors assessed a panel of 27 SASPs that were previously identified as markers associated with mobility disability [17]. Among these, higher plasma levels of myeloperoxidase (MPO) and Membrane metalloprotease-7 (MMP7) and reduced levels of MMP1 reportedly led to increased risk of MCI in older adults. Importantly, MPO and MMP7 were longitudinally associated with future MCI (24 months later), underscoring their predictive potential. These markers had been previously reported to be associated with different neurological disorders including AD. However, the current study points to a potential mechanistic connection with cellular senescence and SASP as players in development of MCI. If certain cases of MCI are driven by cellular senescence, a possibility that needs to be further explored, then senotherapuetic interventions may offer a novel therapeutic opportunity.

This study brings to light a potential role of senescent cells in progression of MCI and further expands on the health domains where SASPs might function as diagnostic or prognostic markers. Additionally, it provides novel markers that should be monitored in senolytic trials such as SToMP-AD to test the effectiveness of senolytics like quercetin and dasatinib in MCI and AD patients [18]. Monitoring SASP responses to senotherapuetics will be useful in evaluating their potential for monitoring treatment efficacy.

In future studies, given the ever-increasing size and complexity of SASP, exploring a more expansive list of SASP-based biomarkers generated by large-scale discovery-based approaches like mass spectrometry will be a valuable pursuit. Together with machine learning, these approaches may help identify longitudinal association of the markers with clinical phenotypes to help identify robust and prognostic biomarkers or biomarker signatures. Although serum proteomic studies can be challenging due to the high dynamic range of plasma proteins with SASPs particularly being low abundant, recent advances in technology, particularly those applying nanoparticle based enrichment, have allowed robust and unbiased identification and quantification of upwards of a thousand SASP markers in serum from aging cohorts [4,19]. Another challenge of plasma proteomic studies, as noted by the authors, is that the proteins in the SASP can be produced by numerous cell types, and SASP associations with MCI may be difficult to detect, possibly overshadowed by their strong associations with declines in mobility disability or other phenotypes observed in the study population. Therefore, increasingly tissue- and phenotype-specific markers may be needed. Senescent cells and SASP are known to be heterogeneous across different cell types, thus challenging efforts to attribute biomarker changes in traditional SASP markers in circulation to senescence in any given cell type(s). Further characterization of senescent cells from diverse cell types that include cells of the brain (neurons, astrocytes, glia, oligodendrocytes) and their secretomes may be helpful in generating senotype-specific (senescent cell subtype-specific) signatures with greater sensitivity and specificity to MCI and related outcomes. Recent demonstration of organ-specific proteomic signatures and their predictive potential for AD highlight the potential value of tissue-specific signatures in circulation [20]. While there is no shortage of work remaining for the discovery, generation, and validation of increasingly specific SASP-based biomarkers of cognitive decline, this important study provides some of the first candidates to explore and sets us on a path to discover others.

## Declaration of competing interest

The authors declare that they have no known competing financial interests or personal relationships that could have appeared to influence the work reported in this paper.

## References

[1] Basisty N., Kale A., Jeon O.H., Kuehnemann C., Payne T., Rao C. (2020). A proteomic atlas of senescence-associated secretomes for aging biomarker development. PLoS Biol.

[2] Tanaka T., Biancotto A., Moaddel R., Moore A.Z., Gonzalez-Freire M., Aon M.A. (2018). Plasma proteomic signature of age in healthy humans. Aging Cell.

[3] Tanaka T., Basisty N., Fantoni G., Candia J., Moore A.Z., Biancotto A. (2020). Plasma proteomic biomarker signature of age predicts health and life span. eLife.

[4] Olinger B.A.-O., Banarjee R.A.-O., Dey A., Tsitsipatis D., Tanaka T., Ram A. (2024). A plasma proteomic signature links secretome of senescent monocytes to aging- and obesity-related clinical outcomes in humans. MedRxiv.

[5] Chinta S.J., Woods G., Demaria M., Rane A., Zou Y., McQuade A. (2018). Cellular Senescence is induced by the environmental neurotoxin paraquat and contributes to neuropathology linked to Parkinson’s disease. Cell Reports.

[6] Bussian T.J., Aziz A., Meyer C.F., Swenson B.L., van Deursen J.M., Baker D.J. (2018). Clearance of senescent glial cells prevents tau-dependent pathology and cognitive decline. Nature.

[7] Dehkordi S.K., Walker J., Sah E., Bennett E., Atrian F., Frost B. (2021). Profiling senescent cells in human brains reveals neurons with CDKN2D/p19 and tau neuropathology. Nature Aging.

[8] Musi N., Valentine J.M., Sickora K.R., Baeuerle E., Thompson C.S., Shen Q. (2018). Tau protein aggregation is associated with cellular senescence in the brain. Aging Cell.

[9] Bhat R., Crowe E.P., Bitto A., Moh M., Katsetos C.D., Garcia F.U. (2012). Astrocyte senescence as a component of Alzheimer’s disease. PLoS One.

[10] Yang J., Zhou Y., Wang T., Li N., Chao Y., Gao S. (2024). A multi-omics study to monitor senescence-associated secretory phenotypes of Alzheimer’s disease. Ann Clin Transl Neurol.

[11] Giau V.A.-O., Bagyinszky E.A.-O., An S.S.A. (2019). Potential fluid biomarkers for the diagnosis of mild cognitive impairment. Int J Mol Sci.

[12] Hou Z.A.-O., Sun A.A.-O., Li Y., Song X., Liu S., Hu X. (2024). What are the reliable plasma biomarkers for mild cognitive impairment? A clinical 4D proteomics study and validation. Mediators Inflammation.

[13] Song F., Poljak A., Smythe G.A., Sachdev P. (2009). Plasma biomarkers for mild cognitive impairment and Alzheimer’s disease. Brain Res Rev.

[14] Salech F., SanMartín C.D., Concha-Cerda J., Romero-Hernández E., Ponce D.P., Liabeuf G. (2022). Senescence markers in peripheral blood mononuclear cells in amnestic mild cognitive impairment and Alzheimer’s disease. Int J Mol Sci.

[15] Mielke M.M., Fielding R.A., Atkinson E.J., Aversa Z., Schafer M.J., Cummings S.R. (2025). Biomarkers of cellular senescence predict risk of mild cognitive impairment: results from the lifestyle interventions for elders (LIFE) study. J Nutr Health Aging.

[16] Fielding R.A., Rejeski W.J., Blair S., Church T., Espeland M.A., Gill T.M. (2011). The lifestyle interventions and independence for elders study: design and methods. J Gerontol A.

[17] Fielding R.A., Atkinson E.J., Aversa Z., White T.A., Heeren A.A., Achenbach S.J. (2022). Associations between biomarkers of cellular senescence and physical function in humans: observations from the lifestyle interventions for elders (LIFE) study. GeroScience.

[18] Gonzales M.M., Garbarino V.R., Kautz T., Palavicini J.A.-O., Lopez-Cruzan M.A.-O., Dehkordi S.A.-O. (2023). Senolytic therapy to modulate the progression of Alzheimer’s Disease (SToMP-AD) - Outcomes from the first clinical trial of senolytic therapy for Alzheimer’s Disease. Res Square.

[19] Dey A.K., Banarjee R., Boroumand M., Rutherford D.V., Strassheim Q., Nyunt T. (2023). Translating senotherapeutic interventions into the clinic with emerging proteomic technologies. Biology (Basel).

[20] Oh H.S.-H., Rutledge J., Nachun D., Pálovics R., Abiose O., Moran-Losada P. (2023). Organ aging signatures in the plasma proteome track health and disease. Nature.

